# Microwave synthesis, crystal structure, antioxidant, and antimicrobial study of new 6-heptyl-5,6-dihydrobenzo[4,5]imidazo[1,2-*c*]quinazoline compound

**DOI:** 10.1186/s13065-018-0509-z

**Published:** 2018-12-20

**Authors:** Hiba Ali Hasan, Emilia Abdulmalek, Mohd Basyaruddin Abdul Rahman, Khozirah Binti Shaari, Bohari Mohd. Yamin, Kim Wei Chan

**Affiliations:** 1 Integrated Chemical BioPhysics Research, Universiti Putra Malaysia, 43400 UPM Serdang, Selangor Malaysia; 2 Department of Chemistry, Faculty of Science, Universiti Putra Malaysia, 43400 UPM Serdang, Selangor Malaysia; 3 Department of Pharmacognosy and Medicinal Plants, College of Pharmacy, Mustansiriyah University, Baghdad, Iraq; 4 Laboratory of Natural Products, Institute of Bioscience, Universiti Putra Malaysia, 43400 Serdang, Selangor Malaysia; 5 Faculty of Science and Technology, Universiti Sains Islam Malaysia, 71800 Nilai, Negeri Sembilan Malaysia; 6 Laboratory of Molecular Biomedicine, Institute of Bioscience, Universiti Putra Malaysia, 43400 Serdang, Selangor Malaysia

**Keywords:** Single crystal, Antioxidant, ABTS, DPPH, Dihydrobenzo[4,5]imidazo[1,2-*c*]quinazoline

## Abstract

**Background:**

Although the development of antibiotic and antioxidant manufacturing, the problem of bacterial resistance and food and/or cosmetics oxidation still needs more efforts to design new derivatives which can help to minimize these troubles. Benzimidazo[1,2-*c*]quinazolines are nitrogen-rich heterocyclic compounds that possess many pharmaceutical properties such as antimicrobial, anticonvulsant, immunoenhancer, and anticancer.

**Results:**

A comparative study between two methods, (microwave-assisted and conventional heating approaches), was performed to synthesise a new quinazoline derivative from 2-(2-aminophenyl)-1*H*-benzimidazole and octanal to produce 6-heptyl-5,6-dihydrobenzo[4,5]imidazo[1,2-*c*]quinazoline (OCT). The compound was characterised using FTIR, ^1^H and ^13^C NMR, DIMS, as well as X-ray crystallography. The most significant peak in the ^13^C NMR spectrum is C-7 at 65.5 ppm which confirms the cyclisation process. Crystal structure analysis revealed that the molecule grows in the monoclinic crystal system P2_1_/n space group and stabilised by an intermolecular hydrogen bond between the N1–H1A…N3 atoms. The crystal packing analysis showed that the molecule adopts zig-zag one dimensional chains. Fluorescence study of OCT revealed that it produces blue light when expose to UV-light and its’ quantum yield equal to 26%. Antioxidant activity, which included DPPH^·^ and ABTS^·+^ assays was also performed and statistical analysis was achieved via a paired T-test using Minitab 16 software with P < 0.05. Also, the antimicrobial assay against two Gram-positive, two Gram-negative, and one fungus was screened for these derivatives.

**Conclusions:**

Using microwave to synthesise OCT have drastically reduced reaction time, and increased yield. OCT show good antioxidant activity in one of the tests and moderate antimicrobial activity.

**Electronic supplementary material:**

The online version of this article (10.1186/s13065-018-0509-z) contains supplementary material, which is available to authorized users.

## Background

**Fig. 1:**
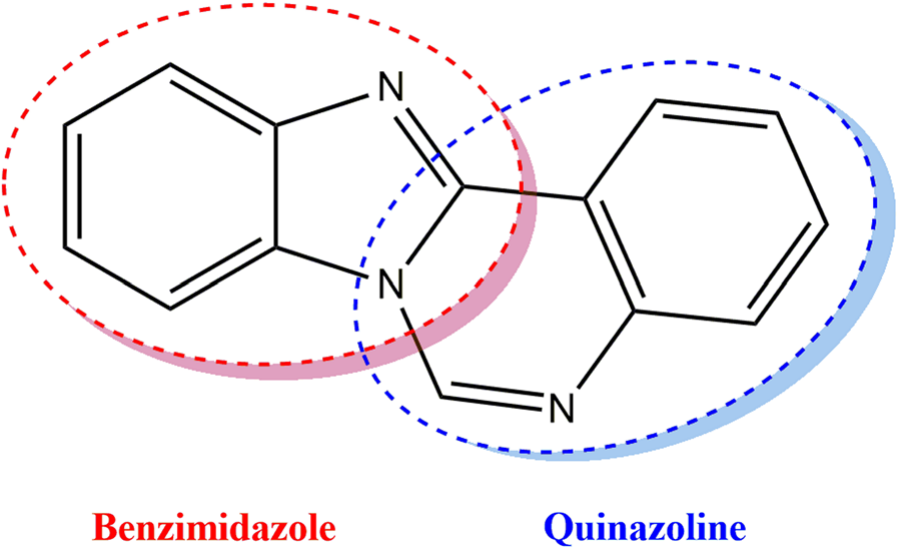
Benzimidazoquinazoline scaffold

Nitrogen-comprising heterocyclic compounds have attracted the interest and attention of many researchers within the medicinal chemistry field over recent years. One of which is the benzimidazo[1,2-*c*]quinazoline nucleus, which is formed from the fusion of benzimidazole to quinazoline bioactive systems (Fig. 1). Literatures revealed that benzimidazoquinazolines possess many distinctive therapeutic properties such as antitumor, anticonvulsant, antioxidant, antimicrobial, antiviral, and as potent imunosuppressors [1–5].

Free radicals and various reactive oxygen or nitrogen species are produced either exogenously from pollution, radiation and food, or endogenously inside the human body from metabolic pathways, leading to oxidative stress. Oxidative stress is the primary cause of many disorders including atherosclerosis, cancer, diabetes, and ageing [6]. Compounds which can scavenge free radicals can, therefore, contribute towards the protection and prevention of these illnesses [7]. Hence, the need for new antioxidants is increasing to solve these problems.

Furthermore, bacterial infections have become a serious threat after many decades of treating the first patient with antibiotic. That is because of the fast increasing in bacterial resistance which become prevalent all over the world. Bacterial resistance to antibiotic is a result of overuse and misuse of these drugs [8]. Therefore, there is continuous need for exploration new medication.

Attempting to solve the said problems, chemists and pharmacists have tried for years to synthesis new nitrogen-comprising compounds which are known for their biological activities. Nevertheless, the problem of using organic solvent in chemical routes presents a significant threat to the environment as it can cause pollution during processing handling, and storage. As a result, many researchers have focused on developing alternative methods and procedures that not only facilitates organic synthesis but also reduces the amounts of solvents. One of these methods uses microwave irradiation to perform organic reactions [9].

Microwave technique to heat organic reactions have been widely discussed and debated within the organic and medicinal chemist community since the publication of the first scientific article in 1986 [10]. In recent years, this fast-moving protocol has been used in many laboratories to synthesise organic materials within a very brief time, resulting in considerable yield, and enhancing pure products. This technique includes direct interaction between the microwave radiation and molecules in the reaction system which dramatically reduces any undesired side-products and increases the yield of the target product [10].

Since 6-heptyl-5,6-dihydrobenzo[4,5]imidazo[1,2-*c*]quinazoline (OCT) is combining skeleton of bioactive quinazoline and benzimidazole nucleolus, it is expected to have some pharmaceutical activities. Also, the literature survey resulted to only one study that have focused on antioxidant activities of benzimidazoquinazoline compounds [11]. Therefore, we report herein the crystal structure, spectroscopic characterisation, antioxidant, and antimicrobial activities of new 6-heptyl-5,6-dihydrobenzo[4,5]imidazo[1,2-*c*]quinazoline resulting from two different synthetic methods.

## Experimental section

### Materials and experimental conditions

The analytical grade chemicals used for this project were commercially available from several suppliers and applied without any additional purification. The glacial acetic acid was supplied from J. T. Baker/USA. The analytical grade methanol and Mueller–Hinton agar were procured from Merck/Germany. The DMSO-*d*_*6*_ for nuclear magnetic resonance was obtained from Merck/Switzerland. The 2-(2-aminophenyl)-1*H*-benzimidazole, octanal, potassium persulfate, 2,2′-azino-bis(3-ethylbenzothiazoline-6-sulfonic acid)diammonium salt (ABTS), (±)-6-hydroxy-2,5,7,8-tetramethylchromane-2-carboxylic acid (Trolox), and 2,2-diphenyl-1-picrylhydrazyl (DPPH) were all supplied from Sigma-Aldrich. Three-angstrom molecular sieves were supplied by Acros Organics/USA and used to dry the solvents.

A 10-mL vial capacity single-mode CEM microwave (USA) along with Synergy software were used to achieve the condensation reaction. An IR Tracer-100 (Shimadzu/Japan) was activated to determine the functional groups applying FTIR analysis and GCMS QP5050A (Shimadzu/Japan) recorded the mass spectrum (DI-MS). JEOL JNM ECA 400 was executed at ambient room temperature to analyse the ^1^H-NMR (400 MHz) and ^13^C-NMR (100 MHz) spectra. A Barnstead Electrothermal/UK instrument was used to measure the melting point, and a Thermo Scientific ELISA reader/UK was used to measure the absorbance of the radical-OCT mixture. A UV–Visible spectrophotometer (UV-1700, Shimadzu/Japan) was operated at ambient room temperature to measure ABTS^·+^ absorbance. An Autopol VI, Automatic Polarimeter manufactured by Rudolph Research Analytical/Hackettstown, NJ, USA was used to measure the optical rotation, and a CHNS instrument (LECO TruSpec Micro CHNS/US) was used to analyse the carbon, hydrogen, and nitrogen percentage contents in the compound. UV-1650 PC (UV–Visible spectrophotometer, SHIMADZU/Japan) was run to measure the UV–Vis absorbance spectra of the studied compounds. Perkin Elmer LS 55 Fluorescence Spectrometer/UK was used to measure emission spectra. Lastly, thin layer chromatography was carried out using silica gel aluminium plates 60 F254 (Merck/Germany).

## Synthesis and characterisation

### Microwave synthesis

The microwave-assisted synthesis was conducted according to Negi et al. [12] with some modifications. In a 10-mL volume microwave vial, octanaldehyde (1.2 mmol, 186 µL) was dissolved in methanol (1 mL) and added dropwise to 2-(2-aminophenyl)-1*H*-benzimidazole (1 mmol, 0.21 g) which was dissolved in 5 mL methanol, followed by addition of two drops of glacial acetic acid. The solution was irradiated in a single-mode benchtop microwave for 5 min at 102 °C, and the reaction was monitored using Synergy software. The TLC was performed to check the progress of the reaction and completion. After 5 min, the vial was cooled to room temperature, dried in a vacuum oven, and washed with hexane to provide the final pure product. The crystals were obtained by slow evaporation of toluene to produce off-white crystalline solid with a premium yield of 91% (0.29 g).

### Conventional heating synthesis

The conventional reflux method was performed according to Kapoor et al. [13] with slight modifications. In a 50-mL round bottom flask, octanaldehyde (1.2 mmol, 186 µL) dissolved in methanol (1 mL) was added dropwise to 2-(2-aminophenyl)-1*H*-benzimidazole (1 mmol, 0.21) which was dissolved in 15 mL hot methanol, followed by addition of two drops of glacial acetic acid. The prepared mixture was refluxed at 95 °C for around 80 min over an oil bath. The reaction progress was monitored every 15 min to check the reaction progression. Next, it was cooled to room temperature after completion as evident by TLC. The target crystals were obtained after vacuum drying, and vigorously washing the crude product with hexane to produce the precipitate which was recrystallised from toluene to furnish off-white, shiny crystals of 77% yield (0.24 g).

#### Characterization of 6-heptyl-5,6-dihydrobenzo[4,5]imidazo[1,2-*c*]quinazoline (OCT)

**Fig. 2:**
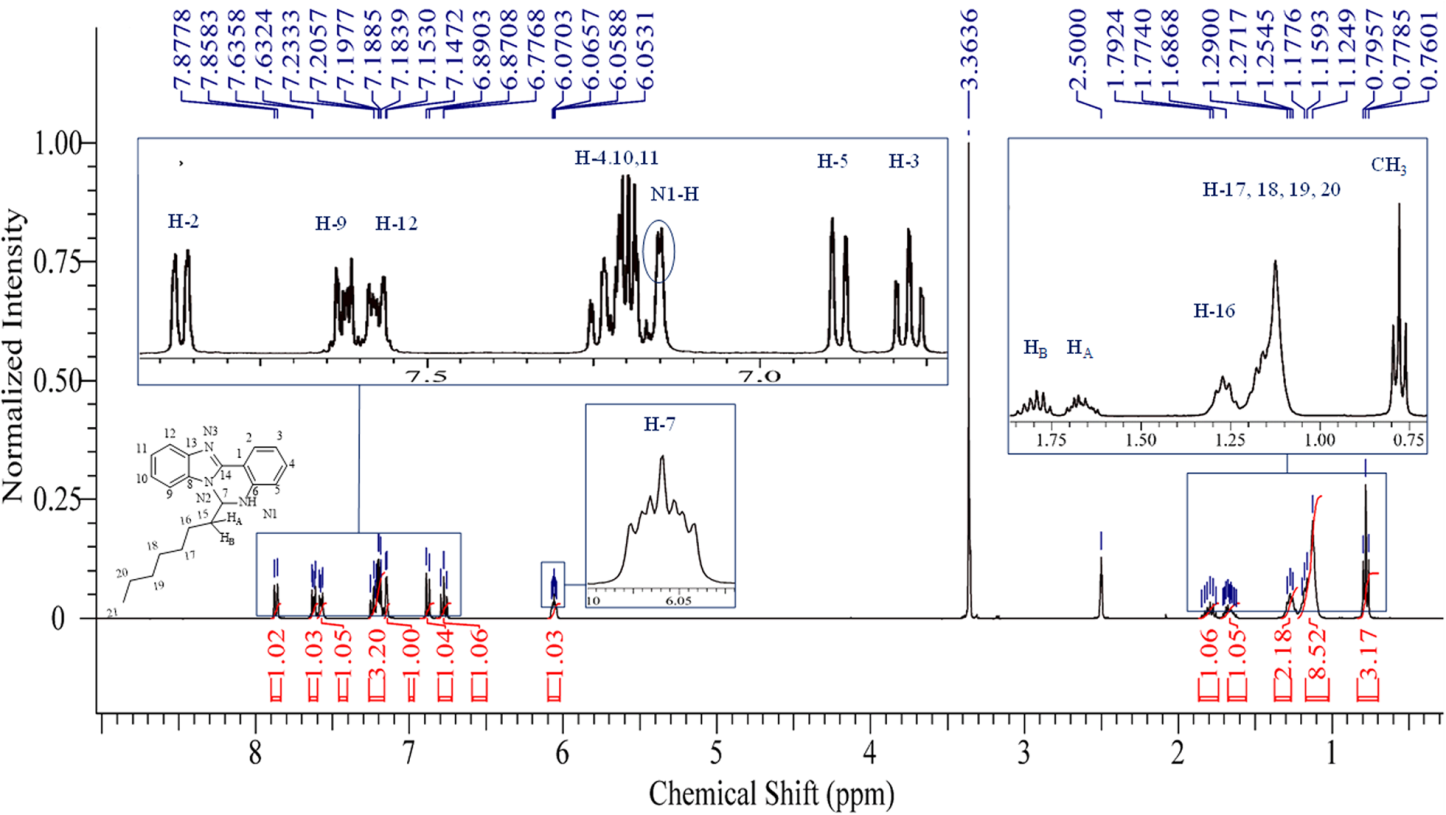
^1^H-NMR spectrum of OCT

**Fig. 3:**
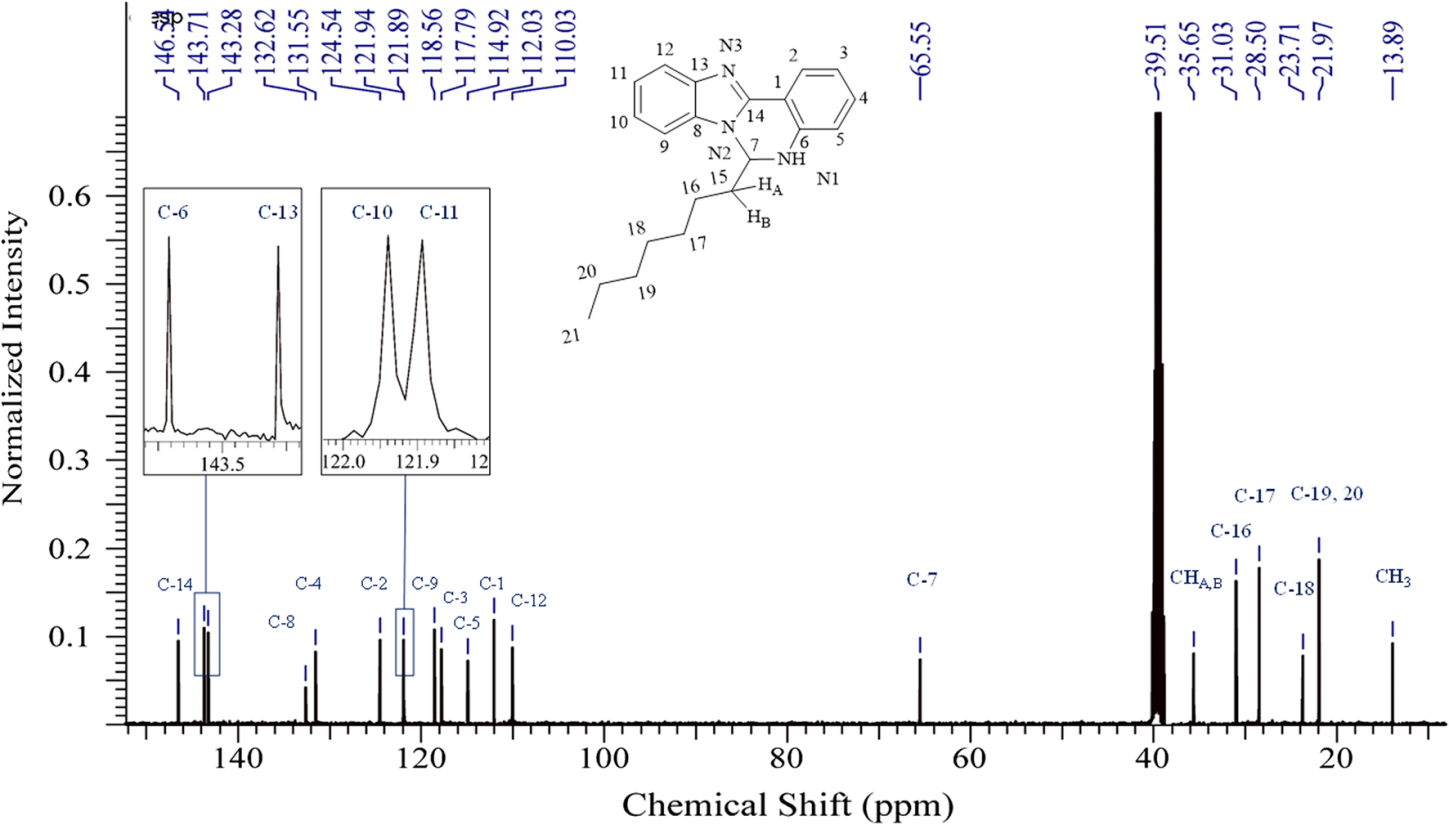
^13^C-NMR of OCT

**Fig. 4:**
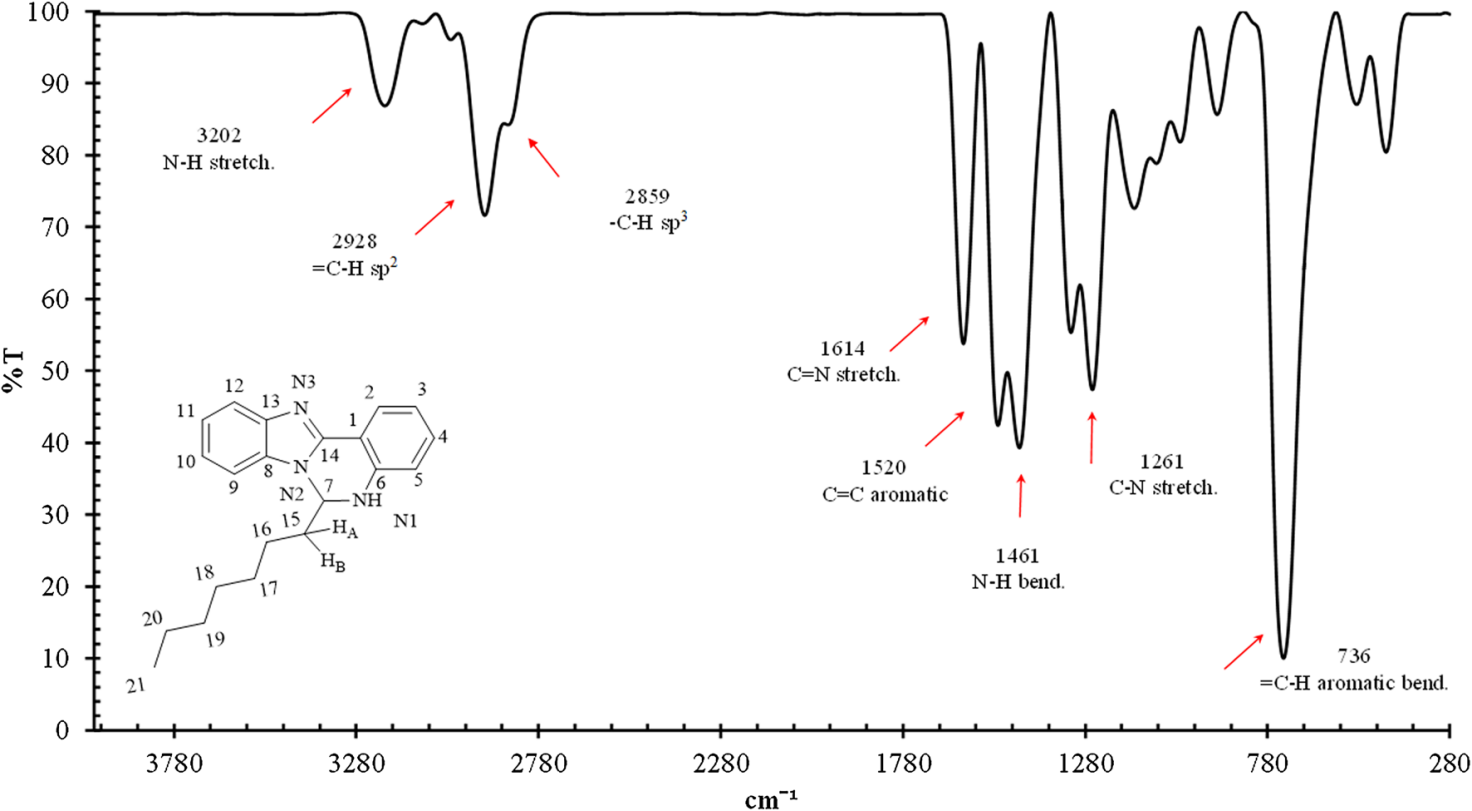
FTIR spectrum of OCT

**Fig. 5:**
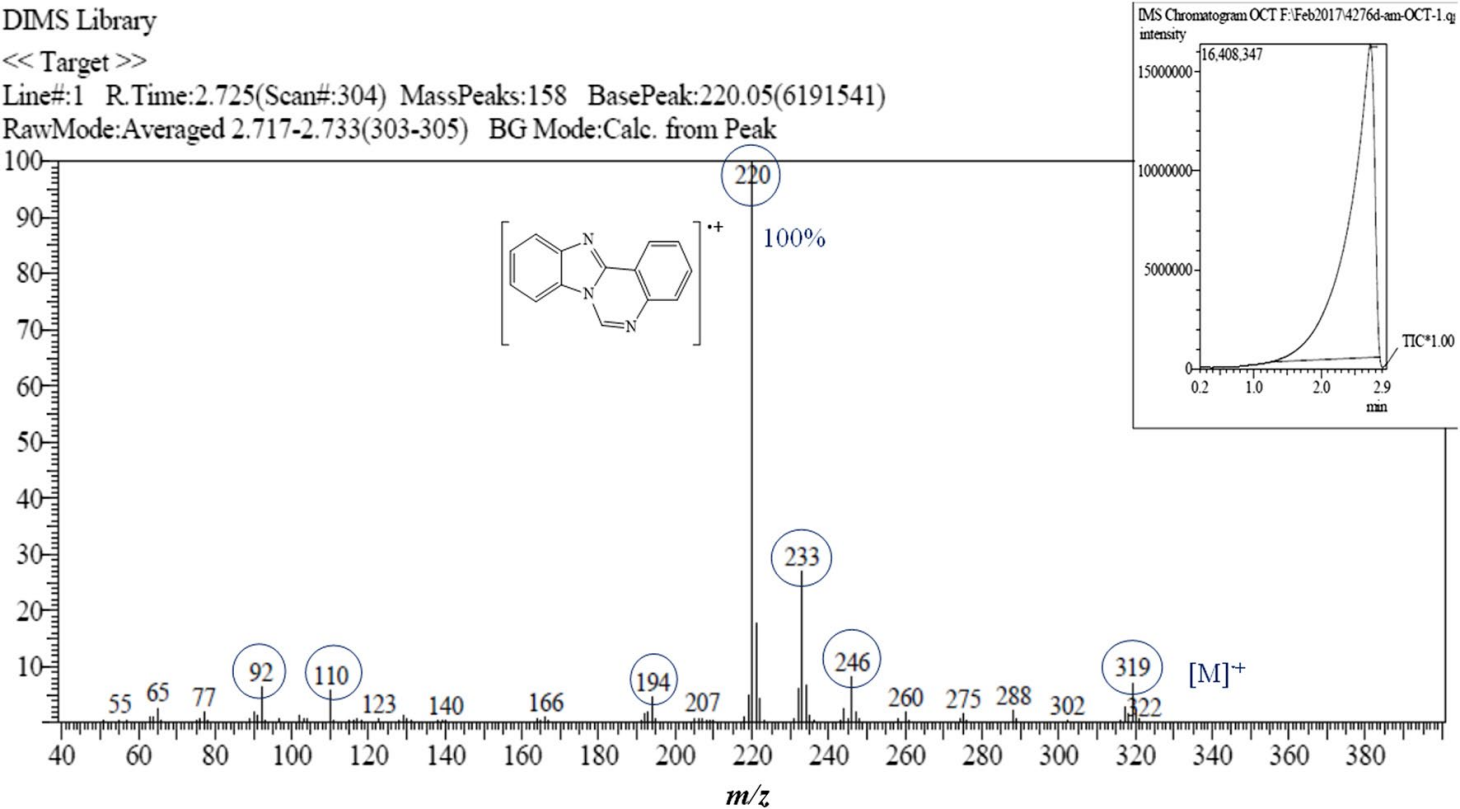
DIMS spectrum of OCT with the main fragments

**Fig. 6:**
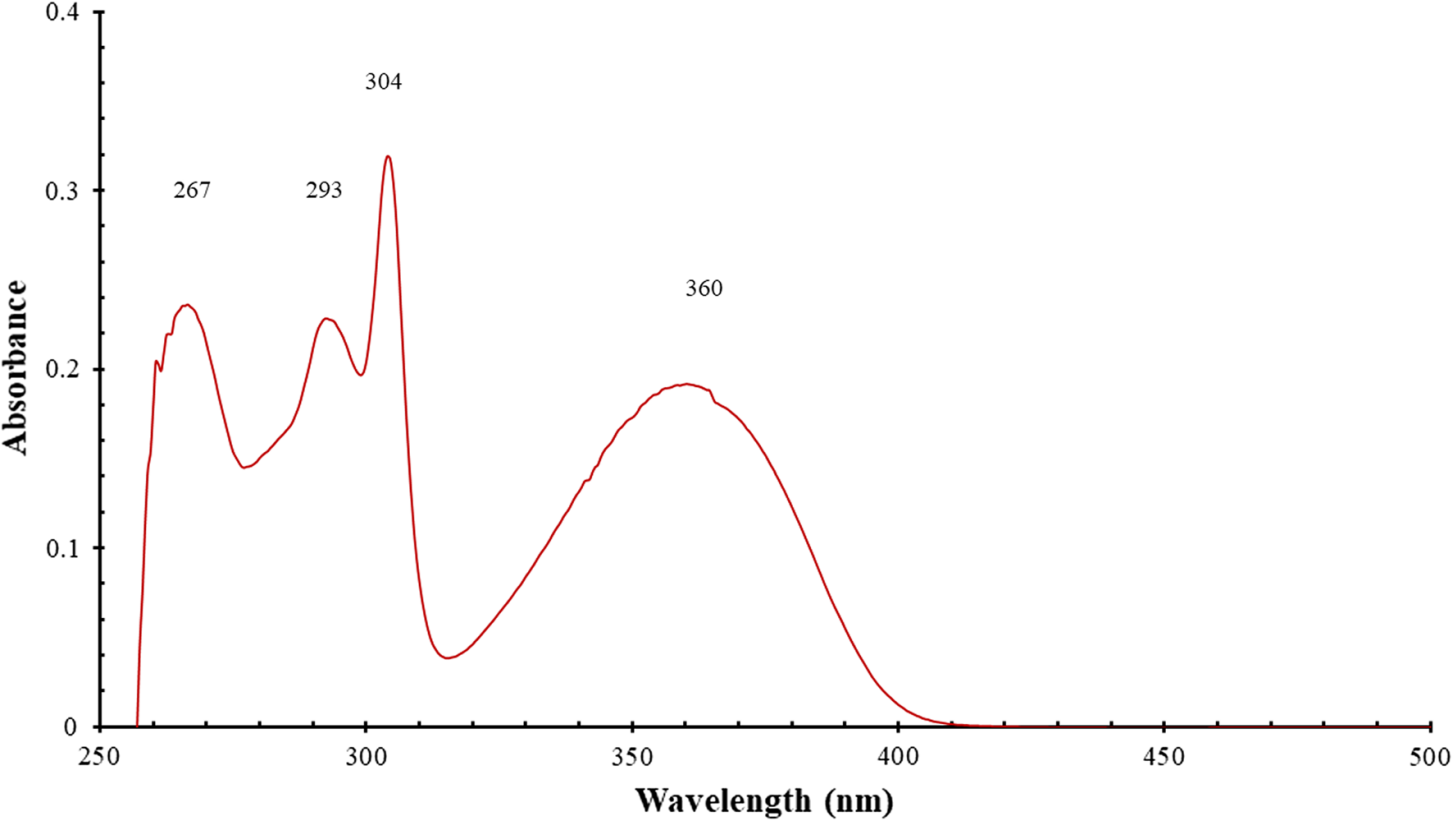
UV–Vis spectrum of OCT

White crystals. M.p.: 116–118 °C; R_f_: 0.50 in hexane: ethyl acetate (2:1) solvent system. $\left[ \alpha \right]_{D}^{20} =$ + 347.3 (c = 0.01, DMSO). FTIR UATR (cm^−1^) ʋmax: 3202 (**N**–**H** stretching), 2928 (–**C**–**H** sp^3^ and =**C**–**H** sp^2^ stretching), 1614 (**C**=**N** stretching), 1520 (**C**=**C** aromatic), 1461 (**N**–**H** bending), 1261 (**C**–**N** stretching), 736 (**C**–**H** bending out of plane for aromatic). ^1^H NMR (400 MHz, *DMSO*-*d*_6_) δ ppm 0.78 (t, *J *= 7.3 Hz, 3H, C**H**_3_**)**, 1.06–1.22 (m, 8H, **H-17**, **18**, **19**, **20**), 1.23–1.32 (m, 2H, **H-16**), 1.61–1.72 (m, 1H, **H**_A_), 1.80 (dt, *J *= 13.8, 7.3 Hz, 1H, **H**_B_), 6.03–6.09 (m, 1H, **H-7**), 6.78 (ddd, *J *= 1.0, 7.9 Hz, 1H, **H-3**), 6.88 (d, *J *= 7.8 Hz, 1H, **H-5**), 7.15 (s, 1H, N1-**H**), 7.17–7.27 (m, 3H, **H-4**, **10**, **11**), 7.55–7.60 (m, 1H, **H-12**), 7.60–7.65 (m, 1H, **H-9**), 7.87 (dd, *J *= 1.4, 7.9 Hz, 1H, **H-2**). ^13^C NMR (100 MHz, DMSO): δc, ppm, 13.8 (**C**H_3_), 21.9 (**C-19**, **20**), 23.7 (**C-18**), 28.5 (**C-17**), 31.0 (**C-16**), 35.6 (**C**H_A_,_B_), 65.5 (**C-7**), 110.0 (**C-12**), 112.0 (**C-1**), 114.9 (**C-5**), 117.7 (**C-3**), 118.5 (**C-9**), 121.8 (**C-11**), 121.9 (**C-10**), 124.5 (**C-2**), 131.5 (**C-4**), 132.6 (**C-8**), 143.2 (**C-13**), 143.7 (**C-6**), 146.5 (**C-14**). MS: DIMS *m/z*: 319 (M^+^, 7%), 246 ([C_16_H_12_N_3_]^+^, 8), 233 ([C_15_H_12_N_3_]^+^, 27), 220 ([C_14_H_10_N_3_]^+^, 100), 194 ([C_13_H_10_N_2_]^+^, 5), 110 ([C_6_H_10_N_2_]^+^, 6), 92 ([C_6_H_6_N]^+^, 6). Anal. Calcd. for C_21_H_25_N_3_: C, 78.96; H, 7.89; N, 13.15%. Found: C, 78.54; H, 7.92; N, 13.19%. UV–Vis in DMSO λ_max_, nm (ɛ, L/mol/cm): 360 (ɛ, 0.191 × 10^4^), 304 (ɛ, 0.319 × 10^4^), 293 (ɛ, 0.228 × 10^4^), 267 (ɛ, 0.236 × 10^4^), (Figs. 2, 3, 4, 5, 6).

### Structure determination by X-ray crystallography analysis

Single crystal X-ray determinations were conducted at Center for Research and Instrumentation (CRIM), Universiti Kebangsaan Malaysia (UKM). A suitable crystal with appropriate size was mounted on a gonio head. Reflection data was collected at 25 °C using (graphite-monochromated Mo Kα radiation, λ = 0.71073 Å) with a photon detector distance of 4 cm and a swing angle of − 30° maximum. The data collected were reduced using the program SAINT [14] and an empirical absorption correction was carried out using SADABS [15]. The structure was solved by direct methods and refined by using the full- matrix least-squares method using the SHELXTL [16] software package. All non-H atoms were anisotropically refined. The hydrogen atoms were located by difference syntheses and refined isotropically. The molecular graphics were created using SHELXTL and MERCURY softwares. PLATON program was used for molecular structure calculation [17]. Atomic scattering factors and anomalous dispersion corrections were taken from the international table for X-ray crystallography.

### Optical activity

Optical rotation of the studied compound was measured for a 0.01 g/100 mL sample concentration dissolved in DMSO at 20 °C, with a 589-nm wavelength. The sample was injected into a 1 dm long polarimeter cell after removing all air bubbles and blanking the instrument. Specific rotation calculated by applying Eq. (1) for the average of five times reading:

1 $$\left[ \alpha \right]_{\lambda }^{T} = \frac{\alpha }{l*c}$$

where, *α* = measured optical rotation. *T* = temperature at measurement process. *λ* = light wavelength in nm, 589 nm using a D line of sodium. *l* = polarimetry cell length in decimetre. *c* = sample concentration in g/mL.

### Elemental analysis

Carbon, hydrogen, and nitrogen percentage analyses were performed to determine the actual ratios of these elements in the OCT sample, comparing them with the calculated ratios.

### Fluorescent study

#### Electronic spectral analysis

UV–Vis absorbance of the studied compounds were measured at room temperature at the concentration of 1 × 10^−4^ M. The samples were dissolved in DMSO at 25 °C and measured at 250–500 nm wavelength. Each spectrum was measured after blanking the instrument with DMSO solvent, and loading the sample to 3 cm^3^ quartz cuvette that has path length of 1 cm. Molar absorptivity calculated by applying Eq. (2):

2 $$\varepsilon \, = \,A/lc$$

where, *ɛ *= The molar absorptivity, L/mol/cm. *A *= the amount of light absorbed by the sample for a given wavelength, without units. *l *= the distance that the light travels through the solution, 1 cm. *c *= the concentration of the absorbing species per unit volume, mole/L.

#### Fluorescence emission study

Fluorescence study was measured at room temperature for 1 × 10^−4^ M for both samples in DMSO and quinine sulfate in 0.1 M solution of H_2_SO_4_ as standard. The quantum yield of all synthesized compounds was obtained from the following method: First, UV–vis absorption spectra for the compounds and quinine sulfate were measured at RT. Then, the emission fluorescence spectra were measured at the low energy excitation wave length which was 360 nm for OCT compound and at 350 for both AMINE and quinine sulfate. Finally, quantum yield was calculated by applying Eq. (3)

3 $$\varPhi Y_{sam} = \varPhi Y_{ref} \frac{{I_{sam} A_{ref} n_{sam}^{2} }}{{I_{ref} A_{sam} n_{ref}^{2} }}$$

where, Subscripts indices “*sam*” and “*ref*” refer to sample and reference, respectively. $\varPhi Y_{ref} = 0.54$ when excited at 350 nm. *I *= Integrated area of emission peak at the excitation wavelength. *A *= UV–vis abortion correction factor which is = $1 - 10^{ - A}$. *n *= refractive index for both water and DMSO.

### Antioxidant activities

#### DPPH^·^ scavenging activity of OCT

The DPPH^·^ scavenging activity of OCT and AMINE was conducted according to Chan et al. [18]. In a 96 well microplate, 50 µL of the diluted OCT sample in DMSO was reacted with 195 μL of 0.2 mM DPPH^·^ (methanolic solution) and kept in a dark ambient room where the mixture was kept for 1 h at 25 °C. Next, using the microplate ELISA reader and at 540 nm, the absorbance was read. The analysis was conducted in triplicate, and the antioxidant activity of both compounds was expressed in mg Trolox equivalent/g sample.

#### ABTS^·+^ scavenging activity of OCT

The ABTS^·+^ scavenging activity of both samples was conducted according to the previous study performed by Chan et al. [19] with some additional modifications. Briefly, ABTS^·+^ was generated by adding 10 mL of 7 mM ABTS to 10 mL of 2.45 mM potassium persulfate and kept in a dark place at room temperature for 24 h. Then, the ABTS^·+^ solution was diluted to the absorbance of 1.40 ± 0.05 at 734 nm with the UV–vis spectrophotometer. Subsequently, 180 μL of ABTS^·+^ solution was added to 20 μL of the OCT sample in a ninety-six well microplate. After 1 h of incubation at room temperature, the absorbance was recorded at 734 nm using a microplate ELISA reader. The analysis was conducted in triplicate, and the scavenging activity of the OCT compound was expressed in mg Trolox equivalent/g sample.

### Statistical analysis

Antioxidant values were expressed as mean ± SD of three replicates for both samples. Statistical analysis was performed by paired T-test using Minitab 16 software with P < 0.05.

### Antimicrobial assay

#### Microbial strain

All the microorganisms used in this study were human clinical strains, provided by the Microbial Culture Collection Unit (UNiCC), Institute of Bioscience, University Putra Malaysia. The microbes strain includes two Gram-positive: *Staphylococcus aureus* ATCC 43300, *Bacillus sublitis* UPMC 1175; two Gram-negative: *Pseudomonas aeruginosa* ATCC 15542, *Salmonella choleraesuis* ATCC 10708; and one fungus: *Aspergillus brasilliensis ATCC 16404*.

#### Antimicrobial activity

The antimicrobial activities of the studied compounds were evaluated using an agar-well diffusion assay [20] with some modifications. Into each of the sterile Petri dishes (Ø 90 mm), 20 mL of molten agar at 45 °C was poured. After the plates were aseptically dried, the agar surface of each plate was streaked using a sterilised cotton swab with the specified microbial strain. Then, with a 5 mm Cork borer diameter, the wells were punctured into the agar. The synthesised compounds were then dissolved in DMSO to produce 100 mg/mL final concentration. Next, 20 μL of the studied samples were loaded into each well, and the plates were incubated invertedly between 30 and 37 °C for 18 and 24 h. or until proper growth had occurred. Once the incubation was completed, the circular inhibition zones were measured using callipers, including the well diameter. The DMSO was used as a negative control while the tetracycline or nystatin was used as a positive control. The experiments were performed in triplicate.

## Results and discussion

### Synthesis

Classical heating, together with microwave heating techniques were undertaken to synthesise the titled crystal (OCT) via the condensation of octanaldehyde with AMINE to compare the reaction time, % yield, purity of the product, and the efficiency of both methods. The results revealed that a microwave-assisted reaction not only produces pure crystals in higher yield but also within a brief reaction time, as summarised in Table 1. Furthermore, the reaction time drastically decreased by 93% when the microwave was applied, and the product percentage yield moderately increased by 14% to produce a very pure product without requiring further purification. From an environmental perspective, this technique is more benign concerning the environment as compared to normal reflux, since the total amount of used methanol was only one-third of the amount used in the conventional heating method.

**Table 1:** Reaction time and % yield of OCT under conventional reflux and microwave irradiation, respectively

OCT	Reaction time, min.			% yield		
	Conventional	MW	% decreased	Conventional	MW	% increase
	75	5	93	77	91	14

**Fig. 7:**
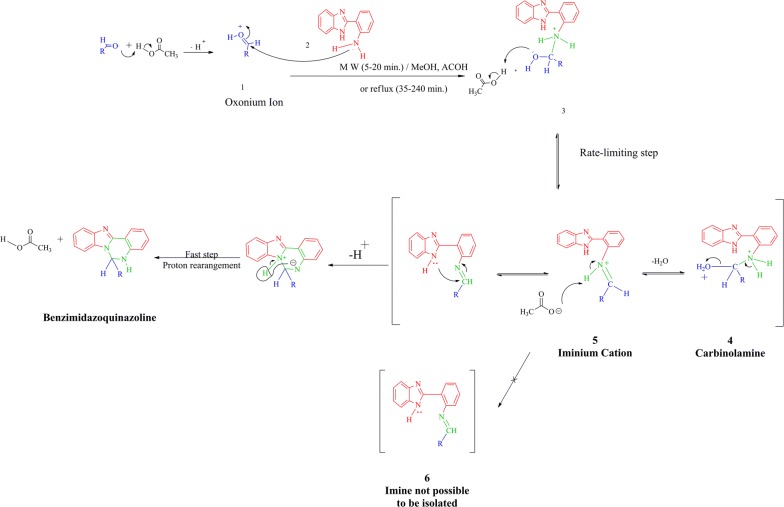
Plausible mechanism for 6-heptyl-5,6-dihydrobenzo[4,5]imidazo[1,2-*c*]quinazoline formation

As illustrated in Fig. 7, the reaction begins by the activation of the carbonyl group of an aldehyde via a protonation step. This is followed by the nucleophilic amine attacking the protonated carbonyl carbon to form the intermediate **3** which was then protonated under acidic reaction conditions to produce carbinolamine intermediate **4**. Notably, this step is considered as a rate-determining step. Meanwhile, carbinolamine is in equilibrium with iminium cation **5** formed by losing a water molecule. Presumably, the imine carbon is quite electrophilic and proceeds to react with the basic secondary amine of the benzimidazole ring to form a new ring following the loss of a proton. Interestingly, the cyclised compound was obtained instead of the expected Schiff base **6** under the same reaction conditions which means that the position of an *ortho*-amino group of the parent amine is the main reason behind the cyclisation process and benzimidazoquinazoline creation.

Seemingly, Schiff base could initially be forming but reacts to create benzimidazoquinazoline, which is applicable for all aldehydes. In the future, the R group in amine can be changed to decrease its’ reactivity to obtain isolate Schiff base compounds.

### Characterisation

The structure of the OCT crystal was confirmed via FTIR, ^1^H and ^13^C NMR, and DIMS and it immediately became apparent by observing the ^1^H, and ^13^C NMR spectra (Figs. 2 and 3) that there was no Schiff base formed, but, a new diazine ring had been formed. Furthermore, there is a new aliphatic multiplet at 6.03–6.09 ppm which belongs to **H-7** of the newly formed ring, and the **N1**–**H** proton appears as a singlet at 7.15 ppm. This, therefore, proved that the cyclisation process rather than Schiff base formation occurred. Moreover, there is no singlet peak around 8.5 to 9 ppm which would belong to the imine proton (–N=**C**–**H**). The ^1^H NMR also displayed four different peaks in the aliphatic area belonging to protons C**H**_3_, **H-17**, **18**, **19** and **20**. The other characteristic peaks are diastereotopic protons **H**_**A**_ and **H**_**B**_ which rose up at different chemical shifts as a multiplet at 1.61–1.72 and doublet of the triplet at 1.80 ppm for **H**_**A**_ and **H**_**B**_ respectively. In the ^13^ C NMR spectrum, the most important peak is **C-7** at 65.5 at the aliphatic area which confirms the cyclisation process and the formation of OCT. Otherwise, there will be a peak around 165 to 170 ppm belonging to carbon (**C**=**N**) of the Schiff base. Figure 3 illustrates the remaining peaks.

The FTIR spectrum of OCT exhibited two medium absorption bands at the 3202 and 2928 cm^−1^ regions corresponding to **N**–**H** and –**C**–**H** sp^2^ stretching, respectively. Also, the band at 2859 and the medium sharp band at 1614 cm^−1^ corresponds to –C–H sp^3^ and **C**=**N** stretching absorptions, respectively. The **C**=**C** aromatic absorption peaks resulted in a medium peak at 1520 cm^−1^, and at 1461 cm ^−1^ the **N**–**H** bending band is observed. Also, the **C**–**N** stretching band appears at 1261 cm ^−1^ and **C**–**H** aromatic out of plane bending at 736 cm ^−1^. Figure 4 summarises all distinctive peaks for the mentioned derivative.

The molecular ion peak was determined for OCT and is equivalent to its molecular weight (C_21_H_25_N_3_ = 319. 44). The peak at 220 *m/z* with 100% intensity is considered as the base peak belonging to the [C_14_H_10_N_3_]^+^ fragment. The remainder of the fragments with their molecular weights is illustrated in Fig. 5.

**Fig. 8:**
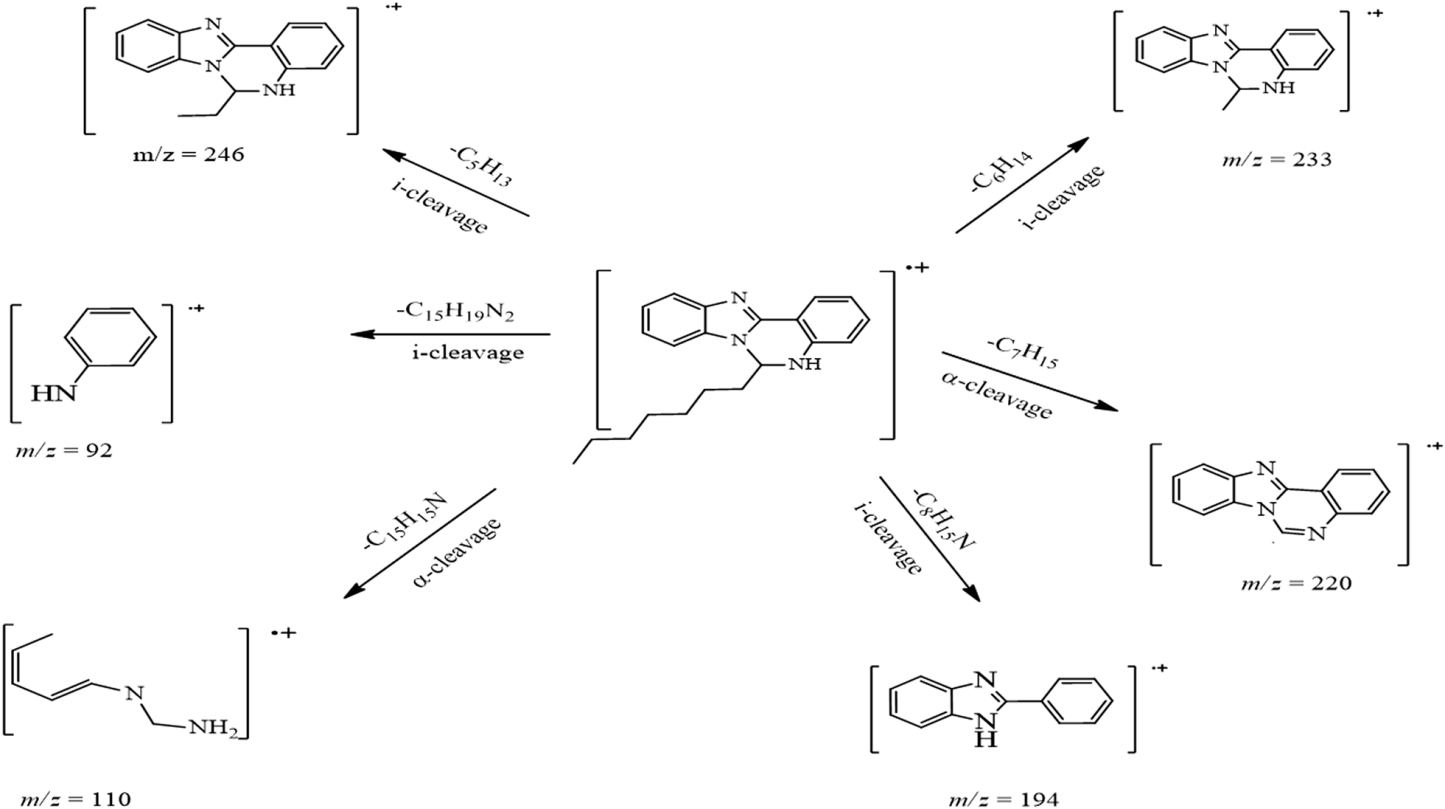
Fragmentation pattern of OCT

As shown in the mass spectrum of the compound 6-heptyl-5,6-dihydrobenzo[4,5]imidazo[1,2-*c*]quinazoline in Fig. 5, the molecular ion peak at 319 *m/z* (7%), which is precisely equal to the calculated molecular weight and the other fragmentation peaks, are also displayed. This molecular ion also underwent α-cleavage to eliminate 6-heptyl moiety to produce a fragment at *m/z* 220 with 100% abundance as a base peak. Further, under the same type of cleavage, a radical ion at *m/z* 110 formed by cutting off C_15_H_15_N moiety. However, under inductive cleavage (i-cleavage), a radical ion at *m/z* 92 was formed via cutting C_15_H_19_N_2_ off, (Fig. 8). Same type of cleavage also occurred to produce a fragment at *m/z* 194 with 4% abundance. Also, both 246 and 233 fragments resulted from the carbon–carbon bond breaking the straight hydrocarbon chain.

### Crystallography study of 6-heptyl-5,6-dihydrobenzo[4,5]imidazo[1,2-*c*]quinazoline (OCT)

6-Hepty5,6-dihydrobenzo[4,5]imidazo[1,2-*c*]quinazoline crystalized in monoclinic system with space group P2_1_/n, a = 9.37 (4), b = 17.14 (5), c = 11.27 (4) Å, α = 90°, β = 101.5 (2)°, ɤ = 90°, z = 4 and volume = 1773 (11) Å^3^. The crystal system and refinement parameters are given in Table 2. The isotopic displacement parameters and structure parameters are given in Additional file 1.

**Table 2:** Refinement of structure and crystal data for 6-heptyl-5,6-dihydrobenzo[4,5]imidazo[1,2-*c*]quinazoline

Identification code	OCT
Empirical formula	C_21_H_25_N_3_
Formula weight	319.20
Temperature	293 (2) K
Wave length	0.71076 Å
Crystal system	Monoclinic
Space group	P2_1_/n
Unit cell dimensions	a = 9.37 (4) Å α = 90°
	b = 17.14 (5) Å β = 101.5 (2)°
	c = 11.27 (4) Å ɣ = 90°
Volume	1773 (11) Å^3^
Z	4
Density (calculated)	1.196 Mg/m^3^
Absorption coefficient	0.071 mm^−1^
F(000)	688
Crystal size	0.500 × 0.430 × 0.270 mm^3^
Theta range for data collection	3.009 to 25.249°
Index ranges	− 11 ≤ h ≤ 11, − 20 ≤ k ≤ 20, − 13 ≤ l ≤ 13
Reflections collected	16,139
Independent reflections	3186 [R(int) = 0.1192]
Completeness to *θ* = 25.243°	99.0%
Refinement method	Full-matrix least-squares on F^2^
Data/restraints/parameters	3186/1/223
Goodness-of-fit on F^2^	1.046
Final R indices [I > 2 sigma (I)]	R1 = 0.1062, wR2 = 0.2552
R indices (all data)	R1 = 0.1858, wR2 = 0.3193
Extinction coefficient	0.015 (4)
Largest diff. peak and hole	0.330 and − 0.297 e Å^−3^
CCDC reference no.	1830213

**Fig. 9:**
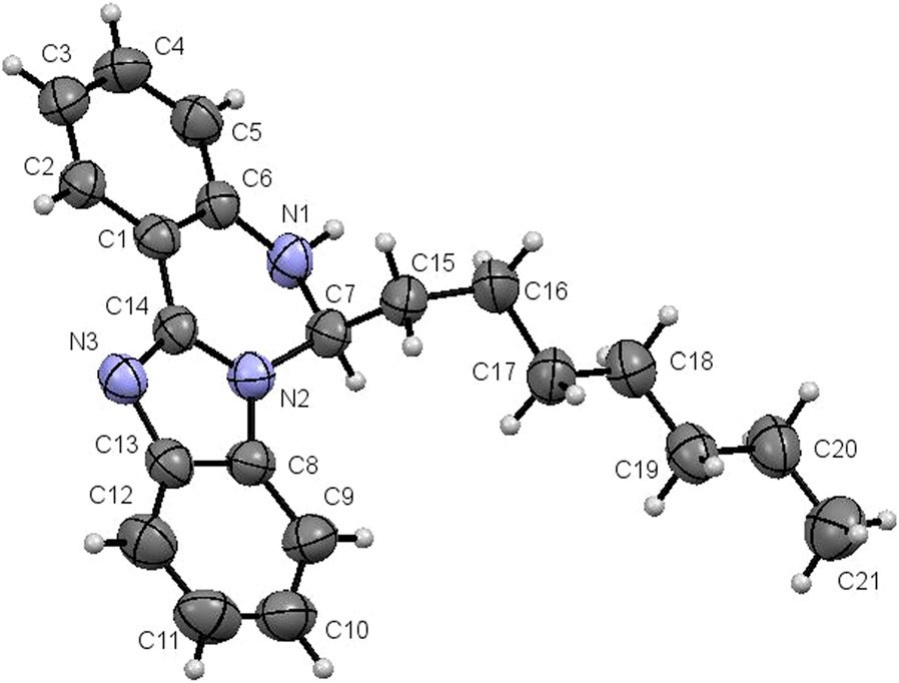
Molecular structure of OCT compound

The molecule is discrete, having only one molecule in the asymmetric unit. The heptyl group is attached to the diazine ring at C7 atom. The molecular structure with the numbering scheme is illustrated in Fig. 9. Notably, the relative configuration at the chiral centre C7 is R which means it is an enantiopure compound.

The benzimidazole ring N2/N3/(C8–C14) is planar with a maximum deviation of 0.012 (5) Å and 0.012 (7) Å for C8 and C11, respectively from the least square plane. The benzene ring (C1–C6) is planar with a maximum deviation of 0.007 (5) Å for C1 from the least square plane. The dihedral angle between the benzimidazole plane and the benzene ring is 7.26 (17)°.

**Fig. 10:**
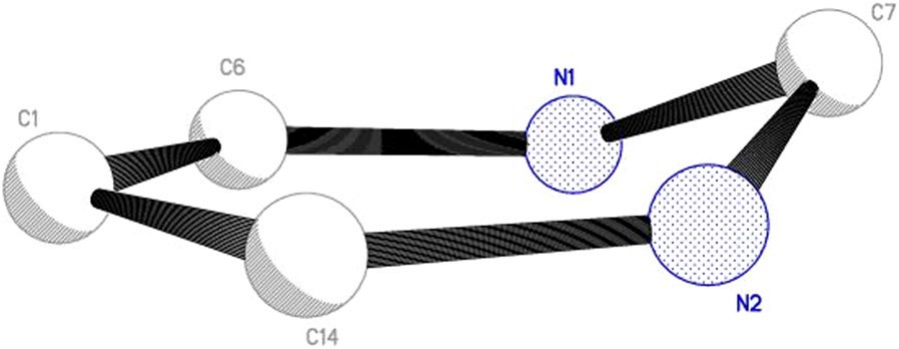
The conformation of diazine ring of OCT

The diazine ring, N1/N2/C1/C6/C7/C14 adopts half-chair conformation with a maximum deviation of 0.209 (5) Å for atom C7 from the least square plane (Fig. 10).

The N3-C14 is 1.318 (7) Å indicating a double bond character while the other bond lengths and angles (Table 3) are in normal ranges and are comparable to those in its analogues of 6-butyl-5,6-dihydrobenzo-[4, 5]imidazo[1,2-*c*]quinazoline [21].

**Table 3:** Selected bond lengths (Å) and angles (°)

Bonds	lengths (Å) and angles (°)
N1–C7	1.439 (7)
N2–C7	1.465 (8)
N2–C14	1.348 (8)
C1–C14	1.455 (8)
C6–C1	1.421 (8)
N1–C6	1.375 (7)
C6–N1–C7	122.2 (5)
N1–C7–N2	107.2 (5)
N1–C7–C15	114.2 (5)
N2–C7–C15	110.9 (5)

**Fig. 11:**
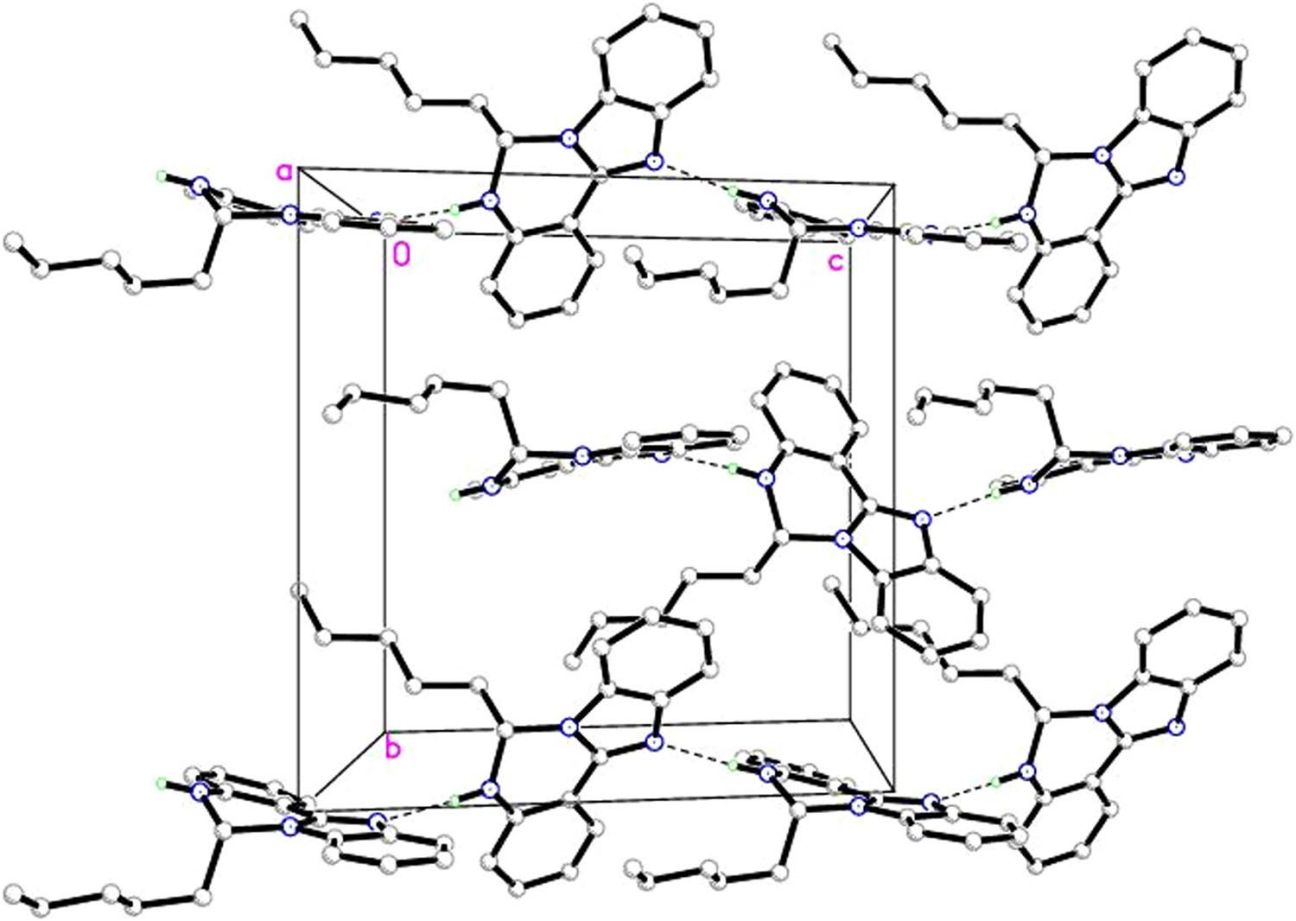
Molecular packing of OCT compound viewed down *a*-axis. All hydrogen atoms except hydrogen bonded are omitted for clarity

In the crystal structure, the molecules are linked by N1–H1A…N3 intermolecular hydrogen bonds (symmetry code as in Table 4) to form zig-zag one dimensional chains (Fig. 11).

**Table 4:** Hydrogen bonds parameters (Å) of OCT compound

Donor—H…acceptor	D–H (Å)	H…A (Å)	D…A (Å)	D—H…A (°)
N1–H1A…N3^*i*^	0.97 (6)	2.08 (6)	3.050 (14)	173 (5)

*i *= 1/2 + x, 1/2 − y, 1/2 + z

### Fluorescent study

The handling and experimental work with this compound unexpectedly disclosed that this compound fluoresces and emits a bright blue colour when exposed to ultraviolet light either from the sun or a UV-lamp. Therefore, it is meaningful if not necessary, to study the fluorescent properties of this compound as a part of the characterisation process which hopefully will expose new potential applications.

### Electronic spectral data

The UV–Vis spectrum of the OCT compound was measured in DMSO solvent at 25 °C and the result exhibited various absorption bands at 267 (ɛ, 0.236 × 10^4^), 293 (ɛ, 0.228 × 10^4^), 304 (ɛ, 0.319 × 10^4^), and 360 (ɛ, 0.191 × 10^4^) nm which are ascribed to π–π^*^ and *n*–π^*^ intramolecular transitions between electronic energy levels. When the OCT compound is exposed to ultraviolet radiation, the electrons are excited and transfer from the highest occupied molecular orbital (HOMO) to the lowest unoccupied molecular orbital (LUMO). The molar absorptivity ε_max_ values (molar extinction coefficient) of this derivative have medium intensities for π → π* transitions which are higher than that of n → π* transition which refers to the higher probability of π electron transitions rather than non-bonding electrons transfer (Fig. 6).

### Emission spectral data

**Fig. 12:**
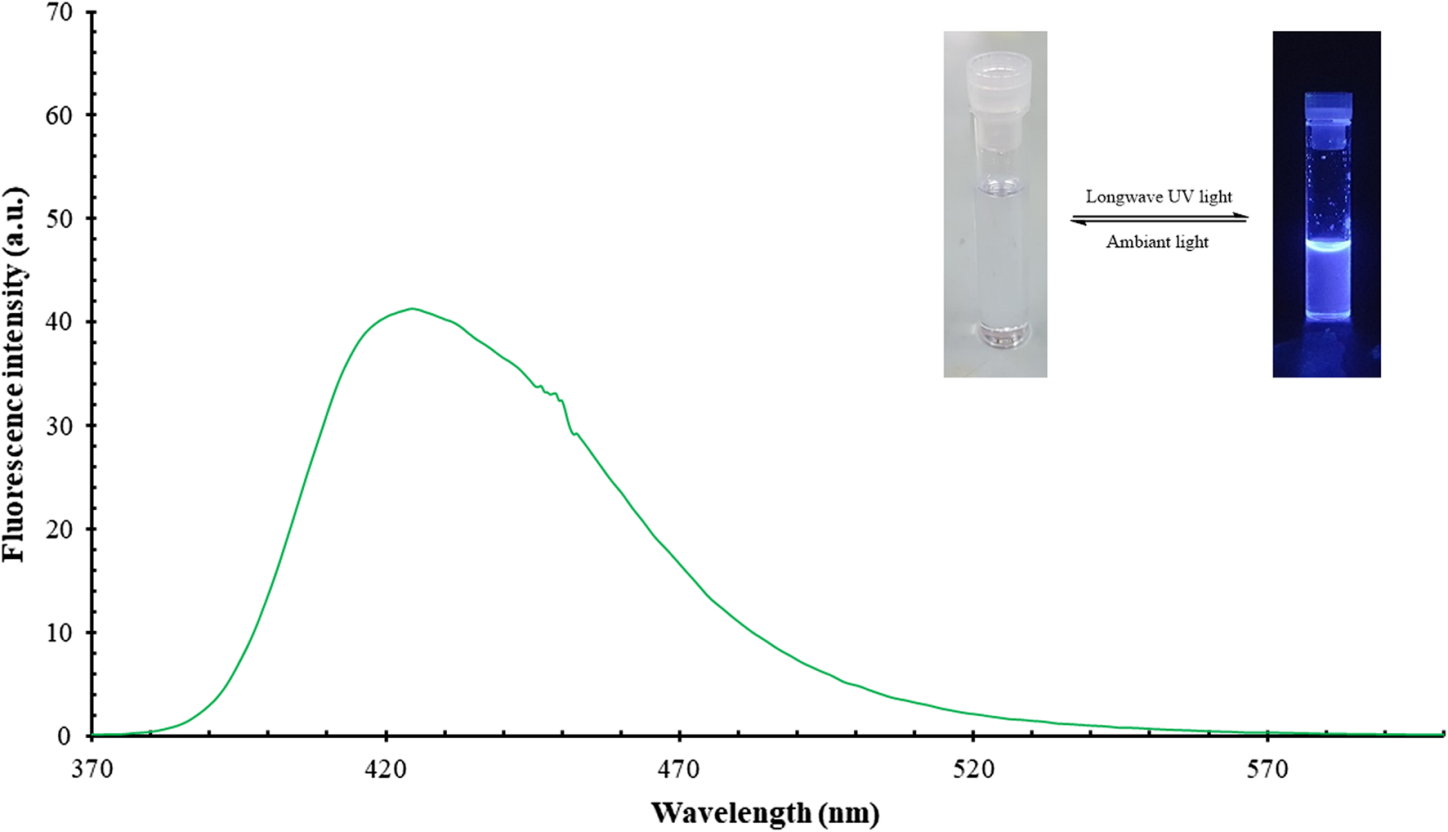
Fluorescence spectrum of OCT compound

**Fig. 13:**
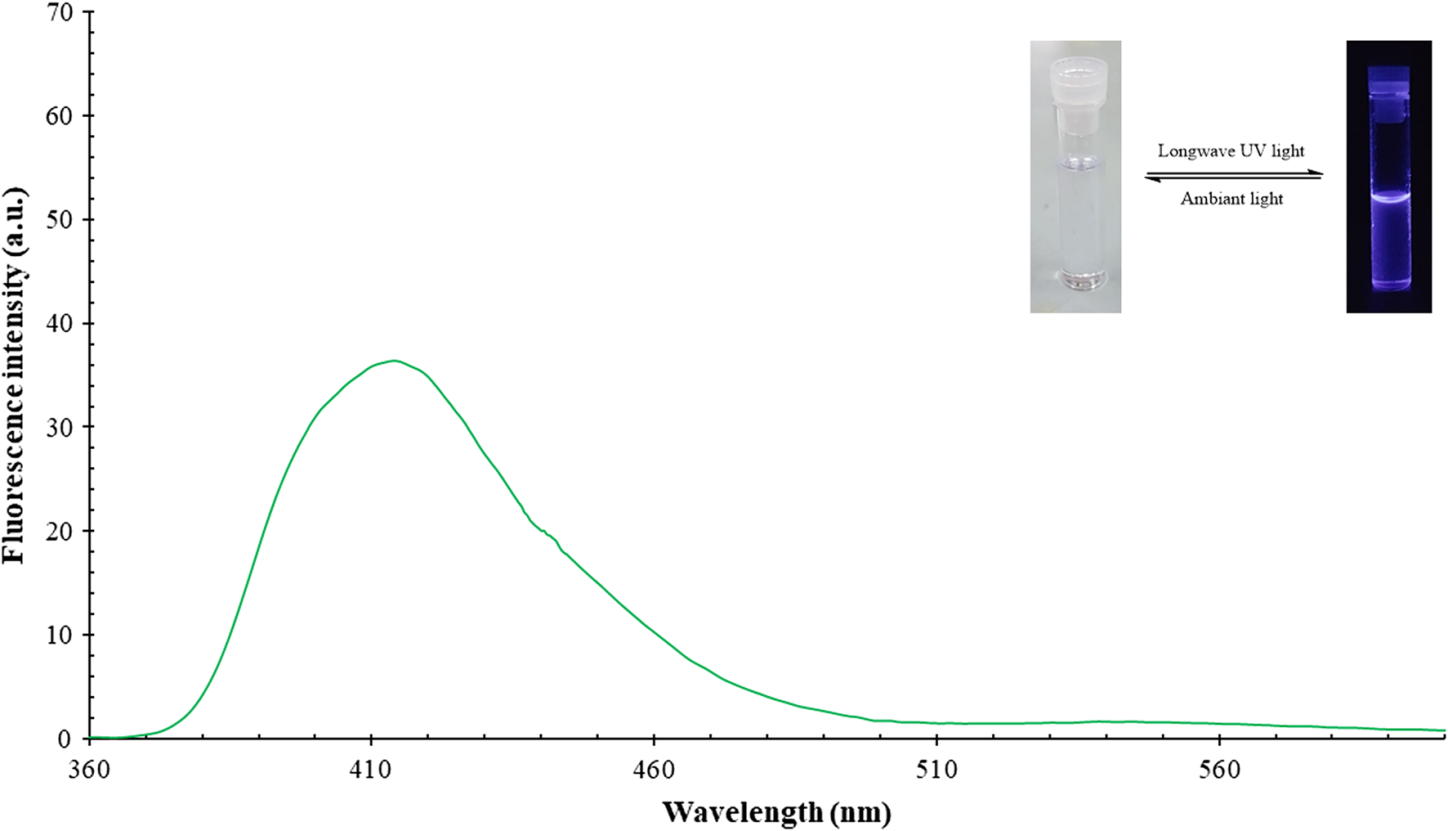
Fluorescence spectrum of AMINE compound

Luminescence is the process that describes the electronic transfer from the excited electronic state to the lower unexcited state. When the emission occurs due to light excitation (usually the UV part of the electromagnetic spectrum), it is called photoluminescence (PL). Notably, fluorescence is one of the members of the luminescence family, and presently, luminescence spectroscopy has wide-ranging applications [22]. Fluorescence spectra of OCT and its’ starting AMINE were measured for a very diluted dimethyl sulfoxide (DMSO) solutions at room temperature. These two solutions are colourless under ambient light but display a very intense blue and purplish-blue colour for OCT and AMINE, respectively under long wave UV light. Also, the PL-spectrum for those compounds gave a wide band in the visible region at 425 and 414 nm for both OCT and AMINE, respectively (Figs. 12 and 13). Table 5 depicts all absorption and emission maxima, stock shifts and quantum yield of these derivatives. Accordingly, the quantum yield (QY) is the ratio of a number of emitted photons to the number of absorbed protons, and its’ evaluation is considered as a key step to characterise fluorescent compounds. The quantum yields for OCT and AMINE were found to be 26% and 13%, respectively compared to 54% of quinine sulphate as the standard. This means that OCT emits around double the amount of light as compared to its’ parent compound.

**Table 5:** Absorption and emission maxima and quantum yields ($\varPhi$) for OCT and AMINE compounds

Compounds	λ_ex_ (nm)	λ_em_ (nm)	Stock shifts (nm)	Quantum yield ($\varPhi$)	Quantum yield (%)
OCT	360	425	65	0.259	26
AMINE	350	414	64	0.129	13
Quinine sulfate	350	458	108	0.54	54

### Antioxidant activities

**Fig. 14:**
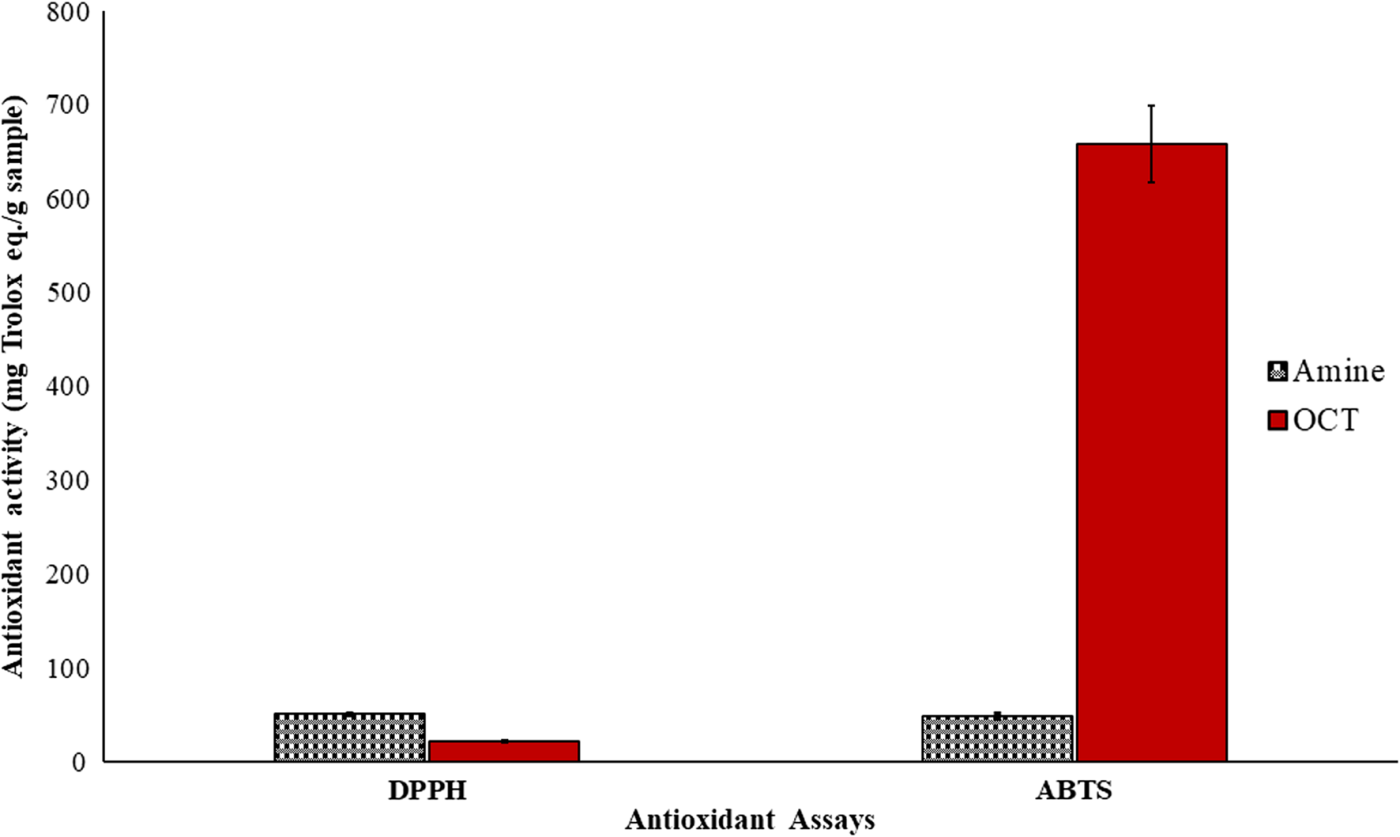
DPPH^·^ and ABTS^·+^ scavenging activity of OCT and AMINE. Results are displayed as mean ± SD (n = 3)

The antioxidant activity of both OCT and the starting material AMINE was next evaluated spectrophotometrically by measuring the ability of both compounds to reduce the reagent radicals, which will be confirmed by decreasing the absorbance of the radical-sample mixture. This can be performed by employing two antioxidant assays, i.e., DPPH^·^ and ABTS^·+^ scavenging activities. The results are illustrated in Fig. 14. Next, the antiradical activity of the OCT and AMINE compounds were evaluated by the reaction of respective crystals with two types of the mentioned stable radicals. The ABTS^·+^ scavenging activities were found to be 658.34 ± 41.01 and 48.61 ± 3.58 mg Trolox eq./g (sample) for the OCT and AMINE samples, respectively, (P < 0.05). The DPPH^·^ scavenging activity of the same samples were 22.27 ± 1.34 and 50.90 ± 1.44 mg Trolox eq./g (sample) for the OCT and AMINE, respectively, (P < 0.05). Therefore, from the results, the ABTS^·+^ scavenging activity of the OCT compound was surprisingly found to be around 30-fold higher than that for DPPH^·^. Indeed, the absence of DPPH^·^ scavenging activity compared to ABTS^·+^ has also been highlighted in many studies [18, 19, 23, 24] and is attributed to the stearic accessibility of the DPPH^·^ radical which is considered as a major hindrance to the chemical reaction. Furthermore, it was found that many small molecules which have better access to the radical site of DPPH^·^, have enhanced scavenging activities as compared to the bulky or rigid molecules which not only slowly react but are also inert in this assay [25]. Notwithstanding, this is also explained in the high reactivity of the starting material AMINE compared to the OCT derivative in this type of test.

### Antimicrobial assay

The preliminary information to test the in vitro antimicrobial activity of the synthesised OCT compound and its initiating material AMINE against five different pathogenic micro-organisms was achieved by applying the agar-well diffusion method. The results confirmed that the range of the inhibition zone mainly depends on the strain of bacteria and fungi. Also, the AMINE compound showed no inhibitory activity for all microorganisms used in this study. In contrast, OCT displayed a moderate inhibitory effect on the selected Gram-positive bacteria with negligible activity against the selected Gram-negative bacteria and *Aspergillus brasiliensis* fungus in this study, as compared to tetracycline and nystatin as a standard antibiotic and anti-fungus, respectively, (refer Table 6). Therefore, it is evident from these results that OCT is a more potent compound compared to its parent because of the bioactive characteristic of the benzimidazoquinazoline compounds [6, 7, 26–28]. Furthermore, the inhibitory activity of OCT was only active against the Gram-positive kind of bacteria, due to the highly resistant Gram-negative bacteria compared to the Gram-positive bacteria. Since the external membrane of the Gram-negative type is rendered with a highly hydrophilic surface, this, therefore, makes it more resistant to antibiotics as compared to Gram-positive bacteria. Also, the negative charge on the Gram-positive wall surface may decrease its resistance to antibacterial derivatives [20]. For the antifungal activity, there is no biological activity for both the studied compounds.

**Table 6:** Antimicrobial activities of studied compounds

Seq.	Compounds concentration in 100 (mg/mL)	Inhibition zone diameter in (mm)^a^				
		Target microbes				
		Gram positive		Gram negative		Fungus
		*Staphylococcus aureus* ATCC 43300	*Bacillus subtilis* UPMC 1175	*Pseudomonas aeruginosa* ATCC 15542	*Salmonella choleraesuis* ATCC 10708	*Aspergillus brasiliensis ATCC 16404*
1	OCT	9	7	–	–	–
2	AMINE	–	–	–	–	–
3	+ve control^b^	28.3	23.6	18.3	25	26
	DMSO (−ve control)	–	–	–	–	–

Values are given as mean of triplicate experiment

–, no inhibition was observed

^a^Diameter of inhibition zones including diameter of 5 mm well

^b^Tetracycline or nystatin in case of antibacterial and antifungal respectively

## Conclusion

The 6-heptyl-5,6-dihydrobenzo[4,5]imidazo[1,2-*c*] quinazoline was successfully synthesised at an excellent yield of 91% using the microwave approach. The FTIR, NMR, and DIMS along with single crystal analysis of titled benzimidazoquinazoline (OCT) confirmed the building structure of this new crystal. The fluorescence study of this compound further disclosed that it fluoresces with double the amount of light compared to the starting AMINE compound. Hence, it could be a potential candidate for further cell imaging applications or single cell level studies for physiological applications. From the antioxidant results, the ABTS^·+^ test revealed higher scavenging activity as compared to the DPPH^·^ test for the same compound. Furthermore, the antimicrobial study of these derivatives demonstrated that OCT is a more active compound as compared to its parent against each of the *Staphylococcus aureus* and *Bacillus subtilis* types of bacteria. Therefore, it could be a good candidate to suppress antibiotic resistant bacteria.

## Additional file

**Additional file 1.** Additional tables.
