# Supplementary material for: Microwave synthesis, crystal structure, antioxidant, and antimicrobial study of new 6-heptyl-5,6-dihydrobenzo[4,5]imidazo[1,2-c]quinazoline compound
Source: Chem Cent J. 2018 Dec 20;12:145. doi: 10.1186/s13065-018-0509-z (PMC6768020; doi:10.1186/s13065-018-0509-z)
Supplement: Supplementary file 1 — Additional file 1. Additional tables. [file 13065_2018_509_MOESM1_ESM.docx]

**Supplementary Data**

**Table S1: Atomic coordinates (x 104) and equivalent isotropic displacement parameters (Å2x 103) for OCT. U (eq) is defined as one third of the trace of the orthogonalized Uij tensor.**

|  | x | y | z | U(eq) |
| --- | --- | --- | --- | --- |
| N(2) | 2741(4) | 3334(2) | 4045(4) | 50(1) |
| N(1) | 3453(5) | 2545(3) | 5752(4) | 56(1) |
| N(3) | 609(5) | 3269(2) | 2754(4) | 56(1) |
| C(9) | 3680(7) | 4590(3) | 3290(6) | 66(2) |
| C(10) | 3256(8) | 5149(4) | 2409(6) | 76(2) |
| C(11) | 1955(9) | 5100(4) | 1597(6) | 81(2) |
| C(12) | 978(7) | 4501(4) | 1635(6) | 74(2) |
| C(13) | 1367(6) | 3932(3) | 2512(5) | 56(1) |
| C(8) | 2715(6) | 3979(3) | 3312(5) | 52(1) |
| C(7) | 4002(5) | 3033(3) | 4906(5) | 50(1) |
| C(6) | 2264(5) | 2067(3) | 5402(4) | 48(1) |
| C(5) | 1956(6) | 1453(3) | 6102(5) | 62(2) |
| C(4) | 733(7) | 1023(3) | 5780(6) | 70(2) |
| C(3) | -271(6) | 1187(3) | 4746(6) | 65(2) |
| C(1) | 1222(5) | 2241(3) | 4334(4) | 48(1) |
| C(14) | 1492(5) | 2936(3) | 3670(4) | 48(1) |
| C(15) | 5059(6) | 2627(3) | 4251(5) | 59(2) |
| C(16) | 6556(6) | 2554(4) | 5064(6) | 68(2) |
| C(17) | 7487(6) | 3287(3) | 5072(5) | 66(2) |
| C(18) | 8819(6) | 3297(4) | 6040(6) | 68(2) |
| C(19) | 9817(6) | 3974(4) | 5972(6) | 71(2) |
| C(20) | 11186(7) | 3985(4) | 6904(6) | 79(2) |
| C(21) | 12172(8) | 4620(4) | 6723(7) | 92(2) |
| C(2) | -9(6) | 1796(3) | 4017(5) | 54(1) |

**Table S2: Bond lengths [Å] and angles [°] for OCT.**

| N(2)-C(14) | 1.348(8) |
| --- | --- |
| N(2)-C(8) | 1.378(7) |
| N(2)-C(7) | 1.465(8) |
| N(1)-C(6) | 1.375(7) |
| N(1)-C(7) | 1.439(7) |
| N(1)-H(1A) | 0.97(6) |
| N(3)-C(14) | 1.318(7) |
| N(3)-C(13) | 1.395(7) |
| C(9)-C(10) | 1.380(9) |
| C(9)-C(8) | 1.386(8) |
| C(9)-H(9A) | 0.9300 |
| C(10)-C(11) | 1.373(11) |
| C(10)-H(10A) | 0.9300 |
| C(11)-C(12) | 1.382(10) |
| C(11)-H(11A) | 0.9300 |
| C(12)-C(13) | 1.385(8) |
| C(12)-H(12A) | 0.9300 |
| C(13)-C(8) | 1.400(9) |
| C(7)-C(15) | 1.518(8) |
| C(7)-H(7A) | 0.9800 |
| C(6)-C(5) | 1.380(8) |
| C(6)-C(1) | 1.421(8) |
| C(5)-C(4) | 1.349(9) |
| C(5)-H(5A) | 0.9300 |
| C(4)-C(3) | 1.372(9) |
| C(4)-H(4A) | 0.9300 |
| C(3)-C(2) | 1.380(8) |
| C(3)-H(3B) | 0.9300 |
| C(1)-C(2) | 1.370(8) |
| C(1)-C(14) | 1.455(8) |
| C(15)-C(16) | 1.520(10) |
| C(15)-H(15A) | 0.9700 |
| C(15)-H(15B) | 0.9700 |
| C(16)-C(17) | 1.529(9) |
| C(16)-H(16A) | 0.9700 |
| C(16)-H(16B) | 0.9700 |
| C(17)-C(18) | 1.484(10) |
| C(17)-H(17A) | 0.9700 |
| C(17)-H(17B) | 0.9700 |
| C(18)-C(19) | 1.502(9) |
| C(18)-H(18A) | 0.9700 |
| C(18)-H(18B) | 0.9700 |
| C(19)-C(20) | 1.486(10) |
| C(19)-H(19A) | 0.9700 |
| C(19)-H(19B) | 0.9700 |
| C(20)-C(21) | 1.469(10) |
| C(20)-H(20A) | 0.9700 |
| C(20)-H(20B) | 0.9700 |
| C(21)-H(21A) | 0.9600 |
| C(21)-H(21B) | 0.9600 |
| C(21)-H(21C) | 0.9600 |
| C(2)-H(2A) | 0.9300 |
| C(14)-N(2)-C(8) | 108.0(5) |
| C(14)-N(2)-C(7) | 124.4(5) |
| C(8)-N(2)-C(7) | 126.5(4) |
| C(6)-N(1)-C(7) | 122.2(5) |
| C(6)-N(1)-H(1A) | 114(3) |
| C(7)-N(1)-H(1A) | 116(3) |
| C(14)-N(3)-C(13) | 104.0(5) |
| C(10)-C(9)-C(8) | 116.3(6) |
| C(10)-C(9)-H(9A) | 121.9 |
| C(8)-C(9)-H(9A) | 121.9 |
| C(11)-C(10)-C(9) | 121.8(6) |
| C(11)-C(10)-H(10A) | 119.1 |
| C(9)-C(10)-H(10A) | 119.1 |
| C(10)-C(11)-C(12) | 122.0(6) |
| C(10)-C(11)-H(11A) | 119.0 |
| C(12)-C(11)-H(11A) | 119.0 |
| C(13)-C(12)-C(11) | 117.6(6) |
| C(13)-C(12)-H(12A) | 121.2 |
| C(11)-C(12)-H(12A) | 121.2 |
| C(12)-C(13)-C(8) | 119.6(6) |
| C(12)-C(13)-N(3) | 129.9(6) |
| C(8)-C(13)-N(3) | 110.6(5) |
| N(2)-C(8)-C(9) | 133.1(6) |
| N(2)-C(8)-C(13) | 104.1(5) |
| C(9)-C(8)-C(13) | 122.7(5) |
| N(1)-C(7)-N(2) | 107.2(5) |
| N(1)-C(7)-C(15) | 114.2(5) |
| N(2)-C(7)-C(15) | 110.9(5) |
| N(1)-C(7)-H(7A) | 108.1 |
| N(2)-C(7)-H(7A) | 108.1 |
| C(15)-C(7)-H(7A) | 108.1 |
| C(5)-C(6)-N(1) | 122.7(5) |
| C(5)-C(6)-C(1) | 117.2(5) |
| N(1)-C(6)-C(1) | 119.9(5) |
| C(4)-C(5)-C(6) | 121.7(6) |
| C(4)-C(5)-H(5A) | 119.1 |
| C(6)-C(5)-H(5A) | 119.1 |
| C(5)-C(4)-C(3) | 121.1(6) |
| C(5)-C(4)-H(4A) | 119.4 |
| C(3)-C(4)-H(4A) | 119.4 |
| C(4)-C(3)-C(2) | 119.2(6) |
| C(4)-C(3)-H(3B) | 120.4 |
| C(2)-C(3)-H(3B) | 120.4 |
| C(2)-C(1)-C(6) | 120.3(5) |
| C(2)-C(1)-C(14) | 123.0(5) |
| C(6)-C(1)-C(14) | 116.5(5) |
| N(3)-C(14)-N(2) | 113.3(5) |
| N(3)-C(14)-C(1) | 127.7(5) |
| N(2)-C(14)-C(1) | 118.8(5) |
| C(7)-C(15)-C(16) | 111.0(5) |
| C(7)-C(15)-H(15A) | 109.4 |
| C(16)-C(15)-H(15A) | 109.4 |
| C(7)-C(15)-H(15B) | 109.4 |
| C(16)-C(15)-H(15B) | 109.4 |
| H(15A)-C(15)-H(15B) | 108.0 |
| C(15)-C(16)-C(17) | 113.1(5) |
| C(15)-C(16)-H(16A) | 109.0 |
| C(17)-C(16)-H(16A) | 109.0 |
| C(15)-C(16)-H(16B) | 109.0 |
| C(17)-C(16)-H(16B) | 109.0 |
| H(16A)-C(16)-H(16B) | 107.8 |
| C(18)-C(17)-C(16) | 114.1(5) |
| C(18)-C(17)-H(17A) | 108.7 |
| C(16)-C(17)-H(17A) | 108.7 |
| C(18)-C(17)-H(17B) | 108.7 |
| C(16)-C(17)-H(17B) | 108.7 |
| H(17A)-C(17)-H(17B) | 107.6 |
| C(17)-C(18)-C(19) | 114.4(5) |
| C(17)-C(18)-H(18A) | 108.7 |
| C(19)-C(18)-H(18A) | 108.7 |
| C(17)-C(18)-H(18B) | 108.7 |
| C(19)-C(18)-H(18B) | 108.7 |
| H(18A)-C(18)-H(18B) | 107.6 |
| C(20)-C(19)-C(18) | 115.6(6) |
| C(20)-C(19)-H(19A) | 108.4 |
| C(18)-C(19)-H(19A) | 108.4 |
| C(20)-C(19)-H(19B) | 108.4 |
| C(18)-C(19)-H(19B) | 108.4 |
| H(19A)-C(19)-H(19B) | 107.4 |
| C(21)-C(20)-C(19) | 112.9(6) |
| C(21)-C(20)-H(20A) | 109.0 |
| C(19)-C(20)-H(20A) | 109.0 |
| C(21)-C(20)-H(20B) | 109.0 |
| C(19)-C(20)-H(20B) | 109.0 |
| H(20A)-C(20)-H(20B) | 107.8 |
| C(20)-C(21)-H(21A) | 109.5 |
| C(20)-C(21)-H(21B) | 109.5 |
| H(21A)-C(21)-H(21B) | 109.5 |
| C(20)-C(21)-H(21C) | 109.5 |
| H(21A)-C(21)-H(21C) | 109.5 |
| H(21B)-C(21)-H(21C) | 109.5 |
| C(1)-C(2)-C(3) | 120.4(6) |
| C(1)-C(2)-H(2A) | 119.8 |
| C(3)-C(2)-H(2A) | 119.8 |

Symmetry transformations used to generate equivalent atoms:

**Table S3: Anisotropic displacement parameters (Å2x 103) for (44). The anisotropic displacement factor exponent takes the form: -2π2[ h2 a*2U11 + ... + 2 h k a* b* U12 ]**

|  | U11 | U22 | U33 | U23 | U13 | U12 |
| --- | --- | --- | --- | --- | --- | --- |
| N(2) | 49(3) | 50(2) | 49(2) | 1(2) | 3(2) | 0(2) |
| N(1) | 45(3) | 70(3) | 50(3) | 8(2) | -2(2) | -2(2) |
| N(3) | 52(3) | 58(3) | 54(3) | 1(2) | 2(2) | 5(2) |
| C(9) | 68(4) | 53(3) | 80(4) | -1(3) | 19(3) | -6(3) |
| C(10) | 87(5) | 58(4) | 89(5) | 8(3) | 33(4) | 1(3) |
| C(11) | 102(6) | 73(4) | 73(4) | 19(3) | 27(4) | 9(4) |
| C(12) | 76(4) | 73(4) | 70(4) | 11(3) | 5(3) | 10(3) |
| C(13) | 55(3) | 51(3) | 60(3) | -3(3) | 10(3) | 9(3) |
| C(8) | 57(3) | 46(3) | 56(3) | 2(2) | 15(3) | 3(2) |
| C(7) | 45(3) | 49(3) | 53(3) | -4(2) | -3(2) | -2(2) |
| C(6) | 44(3) | 51(3) | 49(3) | -3(2) | 10(2) | 3(2) |
| C(5) | 58(4) | 65(3) | 60(3) | 12(3) | 8(3) | 7(3) |
| C(4) | 76(4) | 50(3) | 86(4) | 16(3) | 23(4) | -2(3) |
| C(3) | 57(3) | 52(3) | 87(4) | -4(3) | 17(3) | -3(3) |
| C(1) | 51(3) | 43(3) | 50(3) | -5(2) | 10(2) | 3(2) |
| C(14) | 44(3) | 50(3) | 47(3) | -7(2) | 2(2) | 0(2) |
| C(15) | 47(3) | 57(3) | 72(4) | -3(3) | 10(3) | -2(3) |
| C(16) | 51(3) | 67(4) | 89(4) | 13(3) | 20(3) | 6(3) |
| C(17) | 51(3) | 74(4) | 74(4) | 5(3) | 11(3) | -1(3) |
| C(18) | 59(4) | 72(4) | 77(4) | -1(3) | 17(3) | 6(3) |
| C(19) | 60(4) | 76(4) | 71(4) | -12(3) | 0(3) | 8(3) |
| C(20) | 60(4) | 81(4) | 89(5) | -13(4) | 1(3) | 6(3) |
| C(21) | 81(5) | 88(5) | 99(5) | -7(4) | -5(4) | -12(4) |
| C(2) | 48(3) | 51(3) | 62(3) | -4(3) | 5(2) | -1(2) |

**Table S4: Hydrogen coordinates (x 104) and isotropic displacement parameters (Å2x 10 3) for OCT.**

|  | x | y | z | U(eq) |
| --- | --- | --- | --- | --- |
| H(9A) | 4563 | 4621 | 3838 | 80 |
| H(10A) | 3869 | 5571 | 2364 | 91 |
| H(11A) | 1725 | 5482 | 1002 | 98 |
| H(12A) | 90 | 4481 | 1091 | 89 |
| H(7A) | 4507 | 3477 | 5353 | 61 |
| H(5A) | 2608 | 1332 | 6814 | 74 |
| H(4A) | 568 | 607 | 6266 | 84 |
| H(3B) | -1117 | 892 | 4540 | 78 |
| H(15A) | 5136 | 2922 | 3532 | 70 |
| H(15B) | 4691 | 2112 | 3998 | 70 |
| H(16A) | 7063 | 2115 | 4793 | 82 |
| H(16B) | 6441 | 2444 | 5883 | 82 |
| H(17A) | 7771 | 3334 | 4294 | 80 |
| H(17B) | 6898 | 3739 | 5171 | 80 |
| H(18A) | 9354 | 2817 | 5996 | 82 |
| H(18B) | 8531 | 3308 | 6819 | 82 |
| H(19A) | 10071 | 3973 | 5179 | 85 |
| H(19B) | 9288 | 4453 | 6043 | 85 |
| H(20A) | 10945 | 4039 | 7697 | 94 |
| H(20B) | 11685 | 3490 | 6885 | 94 |
| H(21A) | 13033 | 4602 | 7348 | 139 |
| H(21B) | 11691 | 5112 | 6754 | 139 |
| H(21C) | 12437 | 4562 | 5947 | 139 |
| H(2A) | -674 | 1905 | 3306 | 65 |
| H(1A) | 4180(60) | 2330(30) | 6410(50) | 67(16) |

**Table S5: Torsion angles [°] for OCT.**

| C(8)-C(9)-C(10)-C(11) | -0.3(9) |
| --- | --- |
| C(9)-C(10)-C(11)-C(12) | 1.9(10) |
| C(10)-C(11)-C(12)-C(13) | -1.5(9) |
| C(11)-C(12)-C(13)-C(8) | -0.3(8) |
| C(11)-C(12)-C(13)-N(3) | -179.7(5) |
| C(14)-N(3)-C(13)-C(12) | 179.3(6) |
| C(14)-N(3)-C(13)-C(8) | -0.1(5) |
| C(14)-N(2)-C(8)-C(9) | 178.9(6) |
| C(7)-N(2)-C(8)-C(9) | -13.0(9) |
| C(14)-N(2)-C(8)-C(13) | 1.0(5) |
| C(7)-N(2)-C(8)-C(13) | 169.1(4) |
| C(10)-C(9)-C(8)-N(2) | -179.0(5) |
| C(10)-C(9)-C(8)-C(13) | -1.5(8) |
| C(12)-C(13)-C(8)-N(2) | 180.0(5) |
| N(3)-C(13)-C(8)-N(2) | -0.5(5) |
| C(12)-C(13)-C(8)-C(9) | 1.8(8) |
| N(3)-C(13)-C(8)-C(9) | -178.7(5) |
| C(6)-N(1)-C(7)-N(2) | 37.9(6) |
| C(6)-N(1)-C(7)-C(15) | -85.4(6) |
| C(14)-N(2)-C(7)-N(1) | -33.6(6) |
| C(8)-N(2)-C(7)-N(1) | 160.2(4) |
| C(14)-N(2)-C(7)-C(15) | 91.8(6) |
| C(8)-N(2)-C(7)-C(15) | -74.5(6) |
| C(7)-N(1)-C(6)-C(5) | 162.2(5) |
| C(7)-N(1)-C(6)-C(1) | -23.7(7) |
| N(1)-C(6)-C(5)-C(4) | 175.6(5) |
| C(1)-C(6)-C(5)-C(4) | 1.3(8) |
| C(6)-C(5)-C(4)-C(3) | -1.1(9) |
| C(5)-C(4)-C(3)-C(2) | 1.1(9) |
| C(5)-C(6)-C(1)-C(2) | -1.6(7) |
| N(1)-C(6)-C(1)-C(2) | -176.1(4) |
| C(5)-C(6)-C(1)-C(14) | 174.2(4) |
| N(1)-C(6)-C(1)-C(14) | -0.3(7) |
| C(13)-N(3)-C(14)-N(2) | 0.8(6) |
| C(13)-N(3)-C(14)-C(1) | 176.6(5) |
| C(8)-N(2)-C(14)-N(3) | -1.2(6) |
| C(7)-N(2)-C(14)-N(3) | -169.6(4) |
| C(8)-N(2)-C(14)-C(1) | -177.4(4) |
| C(7)-N(2)-C(14)-C(1) | 14.2(7) |
| C(2)-C(1)-C(14)-N(3) | 4.8(8) |
| C(6)-C(1)-C(14)-N(3) | -170.8(5) |
| C(2)-C(1)-C(14)-N(2) | -179.5(5) |
| C(6)-C(1)-C(14)-N(2) | 4.8(7) |
| N(1)-C(7)-C(15)-C(16) | -77.4(6) |
| N(2)-C(7)-C(15)-C(16) | 161.3(4) |
| C(7)-C(15)-C(16)-C(17) | -83.7(6) |
| C(15)-C(16)-C(17)-C(18) | 168.0(5) |
| C(16)-C(17)-C(18)-C(19) | 173.8(5) |
| C(17)-C(18)-C(19)-C(20) | -177.9(5) |
| C(18)-C(19)-C(20)-C(21) | 174.8(6) |
| C(6)-C(1)-C(2)-C(3) | 1.7(7) |
| C(14)-C(1)-C(2)-C(3) | -173.8(5) |
| C(4)-C(3)-C(2)-C(1) | -1.4(8) |

Symmetry transformations used to generate equivalent atoms:
